# Mycobacterial F_420_H_2_-Dependent Reductases Promiscuously Reduce Diverse Compounds through a Common Mechanism

**DOI:** 10.3389/fmicb.2017.01000

**Published:** 2017-05-31

**Authors:** Chris Greening, Thanavit Jirapanjawat, Shahana Afroze, Blair Ney, Colin Scott, Gunjan Pandey, Brendon M. Lee, Robyn J. Russell, Colin J. Jackson, John G. Oakeshott, Matthew C. Taylor, Andrew C. Warden

**Affiliations:** ^1^Land and Water Flagship, The Commonwealth Scientific and Industrial Research Organisation, ActonACT, Australia; ^2^School of Biological Sciences, Monash University, ClaytonVIC, Australia; ^3^Research School of Chemistry, Australian National University, ActonACT, Australia

**Keywords:** F_420_, redox, biocatalysis, promiscuity, biodegradation, *Mycobacterium*, Actinobacteria

## Abstract

An unusual aspect of actinobacterial metabolism is the use of the redox cofactor F_420_. Studies have shown that actinobacterial F_420_H_2_-dependent reductases promiscuously hydrogenate diverse organic compounds in biodegradative and biosynthetic processes. These enzymes therefore represent promising candidates for next-generation industrial biocatalysts. In this work, we undertook the first broad survey of these enzymes as potential industrial biocatalysts by exploring the extent, as well as mechanistic and structural bases, of their substrate promiscuity. We expressed and purified 11 enzymes from seven subgroups of the flavin/deazaflavin oxidoreductase (FDOR) superfamily (A1, A2, A3, B1, B2, B3, B4) from the model soil actinobacterium *Mycobacterium smegmatis*. These enzymes reduced compounds from six chemical classes, including fundamental monocycles such as a cyclohexenone, a dihydropyran, and pyrones, as well as more complex quinone, coumarin, and arylmethane compounds. Substrate range and reduction rates varied between the enzymes, with the A1, A3, and B1 groups exhibiting greatest promiscuity. Molecular docking studies suggested that structurally diverse compounds are accommodated in the large substrate-binding pocket of the most promiscuous FDOR through hydrophobic interactions with conserved aromatic residues and the isoalloxazine headgroup of F_420_H_2_. Liquid chromatography-mass spectrometry (LC/MS) and gas chromatography-mass spectrometry (GC/MS) analysis of derivatized reaction products showed reduction occurred through a common mechanism involving hydride transfer from F_420_H^-^ to the electron-deficient alkene groups of substrates. Reduction occurs when the hydride donor (C5 of F_420_H^-^) is proximal to the acceptor (electrophilic alkene of the substrate). These findings suggest that engineered actinobacterial F_420_H_2_-dependent reductases are promising novel biocatalysts for the facile transformation of a wide range of α,β-unsaturated compounds.

## Introduction

Industrial biocatalysts are making a substantial impact in the selective synthesis of pharmaceuticals and other specialist chemicals (Nestl et al., 2011; Clouthier and Pelletier, 2012). Enzymes that mediate selective alkene reduction are in particular demand (Stuermer et al., 2007). The most widely investigated of these enzymes are the “old yellow enzyme” family of NAD(P)H-dependent flavoproteins. These often-promiscuous enzymes have been shown to catalyze hydride addition to activated alkene groups of diverse substrates of both natural (e.g., quinones) and synthetic (e.g., cyclohexenones) origin (Williams and Bruce, 2002; Stuermer et al., 2007; Amato and Stewart, 2015). Their catalytic cycle proceeds by hydride transfer from bound FMNH_2_ to the substrate, protonation of the reduced substrate by a conserved tyrosine, and reduction of the cofactor by the external hydride donor NAD(P)H (Fox and Karplus, 1994). Such enzymes are in development as *in vitro* biocatalysts and are critical in several industrial fermentation processes (e.g., levodione synthesis; Stuermer et al., 2007; Amato and Stewart, 2015). Despite these successes, there is still demand for the discovery of novel reductive biocatalysts to provide more flexible platforms for development of specific *in vitro* and *in vivo* syntheses.

Actinobacteria represent a particularly promising source of novel biocatalysts. This phylum includes genera reputed for their biodegradative capacity, notably *Mycobacterium* and *Rhodococcus*, as well as *Streptomyces* strains that are vital sources of natural products (Barka et al., 2016). One reason these organisms are so metabolically versatile is that they synthesize the unusual redox cofactor F_420_ (Greening et al., 2016; Ney et al., 2017). The low standard redox potential (*E*°′ = -340 mV) and obligate two-electron chemistry of F_420_H_2_ means that it can reduce compounds otherwise recalcitrant to activation (Walsh, 1986; Greening et al., 2016). Actinobacteria reduce F_420_ using either the F_420_-dependent glucose-6-phosphate dehydrogenase (Fgd) (Bashiri et al., 2008; Nguyen et al., 2017) or the F_420_-NADP oxidoreductase (Fno) (Eker et al., 1989; Ebert et al., 1999). They subsequently couple the reoxidation of F_420_H_2_ to the hydrogenation of diverse organic compounds. This depends on a suite of F_420_H_2_-dependent reductases from two different superfamilies, the luciferase-like hydride transferases (LLHT superfamily; Ebert et al., 1999; Heiss et al., 2003; Ikeno et al., 2006) and the flavin/deazaflavin oxidoreductases (FDOR superfamily; Taylor et al., 2010; Gurumurthy et al., 2013; Ahmed et al., 2015; Greening et al., 2016). The enzymatic activities and industrial potential of these enzymes have remained largely unexplored.

F_420_H_2_-dependent reductases of the FDOR superfamily have been advocated as particularly promising reductive biocatalysts (Ahmed et al., 2015; Greening et al., 2016). These reductases are abundant in mycobacteria and other Actinobacteria, where they have diverged into at least 14 distinct subgroups (A1–A3, B1–B6, AA1–AA5; Ahmed et al., 2015). While several native functions have been proposed, e.g., menaquinone and biliverdin reduction (Gurumurthy et al., 2013; Ahmed et al., 2015, 2016), the enzymes also mediate promiscuous activities, such as nitroimidazole prodrug activation (Cellitti et al., 2012; Mohamed et al., 2016a,b), biodegradation of furanocoumarins (Taylor et al., 2010; Lapalikar et al., 2012b; Jirapanjawat et al., 2016), and decolorization of triarylmethane dyes (Guerra-Lopez et al., 2007; Jirapanjawat et al., 2016). The findings that these enzymes can reduce such structurally and chemically diverse compounds suggests that they may also have the latent capacity to act upon industrially relevant non-natural chemicals. Mechanistic studies focused on mycobacteria indicate that these enzymes adopt a distinct mechanism from old yellow enzymes that may be relevant for selective synthesis (Greening et al., 2016; Mohamed et al., 2016b). For example, the reduced cofactor is thought to bind the enzyme from the solvent phase and directly mediate hydride addition to the substrate (Mohamed et al., 2016a,b); The cofactor can then be re-reduced *in vitro* and *in vivo* by Fgd (Purwantini and Daniels, 1996; Bashiri et al., 2008). In addition, the proton donor for reduced substrates is a solvent-accessible hydroxonium ion rather than a tyrosine residue (Mohamed et al., 2016b).

In this study, we explored the substrate promiscuity across multiple subgroups of the F_420_H_2_-dependent FDORs to determine their potential value as next-generation biocatalysts. To do this, we tested 11 of these enzymes from the model laboratory organism *Mycobacterium smegmatis* against 47 different substrates, ranging from synthetic building blocks to more complex polycyclic compounds. This revealed that, in common with old yellow enzymes, several of these enzymes can promiscuously reduce diverse cyclic and polycyclic compounds harboring activated alkene groups. Subsequent structural modeling and mechanistic studies suggested that these enzymes reduced these diverse substrates through a common mechanism: regioselective hydride transfer from F_420_H^-^ to the proximal electrophilic alkene group. The considerable promiscuity of these enzymes suggests they are promising candidate biocatalysts, but engineering will be required to optimize their rates in industrial processes.

## Materials and Methods

### Recombinant Protein Expression and Purification

Eleven F_420_H_2_-dependent reductases of the FDOR superfamily (MSMEG loci 5998, 2850, 2027, 5030, 6325, 3380, 0048, 6848, 6526, 5170, 3880; Supplementary Table S1) and the F_420_-reducing glucose-6-phosphate dehydrogenase (Fgd) were recombinantly overexpressed in *E. coli* BL21(DE3). MSMEG_6325, MSMEG_6526, MSMEG_3880 and *fgd* were expressed overnight in modified auto-induction TB2.0 media at 28°C (200 rpm) as previously described (Taylor et al., 2010; Lapalikar et al., 2012b). For the remaining proteins, cells were grown in lysogeny broth (LB) at 37°C (200 rpm) and induced at OD_600_ 0.6 with 0.2% L-arabinose for 2 h. Cells were harvested by centrifugation (10,000 × *g*, 20 min, 4°C), resuspended in lysis buffer (50 mM NaH_2_PO4, 300 mM NaCl, 10 mM imidazole, pH 8.0), and lysed in a EmulsiFlex-C3 homogenizer (ATA Scientific, Australia). The enzymes were purified from soluble extracts by Ni-nitrilotriacetic acid (NTA) affinity chromatography using gravity columns as previously described (Taylor et al., 2010; Ahmed et al., 2015) and stored in elution buffer (50 mM NaH_2_PO_4_ 300 mM NaCl, 250 mM imidazole, pH 8.0) until use in enzymatic assays. The high purity of the proteins was confirmed by running the fractions on NuPAGE Novex 10% Bis-Tris gels (Invitrogen, Australia) at 200 V and staining with Coomassie Brilliant Blue. Protein concentration was determined by measuring absorbance at 280 nm using a NanoDrop ND1000 (NanoDrop Technologies) and calculating concentration with the Beer–Lambert equation. Molar absorption coefficients were calculated for each protein based on amino acid sequences (Supplementary Table S1). F_420_ was extracted, purified, and concentrated from a recombinant F_420_ overexpression strain of *M. smegmatis* mc^2^4517 (Bashiri et al., 2010) as previously described (Isabelle et al., 2002).

### Enzyme Activity Assays

Forty-seven different compounds were sourced from Sigma-Aldrich and dissolved into 1 M working stocks in dimethyl sulfoxide, except hypoxanthine and guanine that were dissolved in 1 M NaOH solution. The structures of the compounds tested are shown in Supplementary Tables S2, S3. Enzymatic assays were performed by spectroscopically monitoring the reoxidation of pre-reduced F_420_H_2_ in the presence of FDOR and substrate. F_420_ was enzymatically reduced to F_420_H_2_ by overnight incubation with 1 μM Fgd and 12 mM glucose 6-phosphate as described (Ahmed et al., 2015). The enzyme was subsequently repurified as described (Ahmed et al., 2015). All reaction mixtures contained degassed Tris buffer [200 mM Tris, 0.1% (w/v) Triton X-100, pH 8.0] sequentially supplemented with 50 μM substrate, 25 μM F_420_H_2_, and 1 μM of the FDOR. Enzyme concentration was decreased to 10 nM for substrates observed to be rapidly reduced, i.e., quinone compounds. Reaction rates were monitored by recording the initial linear increase in 420 nm absorbance using an Epoch 2 Microplate Spectrophotometer (BioTek). All assays were performed at room temperature (approximately 25°C). We only detected significant levels of enzyme-independent, substrate-dependent F_420_H_2_ reoxidation for quinone and arylmethane substrates, at rates that we previously reported (Jirapanjawat et al., 2016). We observed no enzyme-dependent, substrate-independent or spontaneous F_420_H_2_ reoxidation in the timeframe of our assays. Specific activities were calculated after subtracting rates of enzyme-independent F_420_H_2_ reoxidation and were expressed in nmol s^-1^ μmol^-1^ enzyme as previously described (Taylor et al., 2010). The rate of reduction of three of these compounds, namely 1,4-naphthoquinone, 3-cyanocoumarin, and 5,6-dihydro-2*H*-pyran-2-one, was also measured in cofactor-recycling assays. Assays used 100 μM substrate, 0.1 μM enzyme, 10 μM F_420_, 2.5 mM glucose-6-phosphate, and 0.45 μM Fgd. Time course high performance liquid chromatography (HPLC) assays, performed according to published methodologies (Lapalikar et al., 2012b), measured loss of absorbance (at λ_max_) of the substrates at regular time intervals.

### Molecular Docking

Substrates were docked into the previously solved X-ray crystal structures of MSMEG_2027 (1.5 Å resolution; PDB: 4Y9I; Ahmed et al., 2015) and MSMEG_6526 (1.7 Å resolution; PDB: 4KZY; Ahmed et al., 2015). F_420_ was docked into the cofactor-binding pockets based on the cofactor-bound structures of Rv3547 (PDB: 3R5R; Cellitti et al., 2012) and Rv2074 (PDB: 5JAB; Ahmed et al., 2016) respectively. AutoDock Vina was used to computationally dock the substrates into their corresponding enzymes, with enzymes and ligands prepared using AutoDockTools operating with default settings (Morris et al., 2009). The docking results were visualized and analyzed in UCSF Chimera (Pettersen et al., 2004).

### Substrate Reduction and Derivatization

The chemical standards and reaction products of menadione, 3-cyanocoumarin, and 2-cyclohexen-1-one were detected by mass spectrometry. These compounds were reduced by incubating them with the promiscuous F_420_H_2_-dependent reductase MSMEG_2027 for 2 h at 37°C. The assay mixture comprised 100 μM substrate, 10 μM F_420_, 1 μM Fgd, 1 μM MSMEG_2027, and excess G-6-P in either 20 mM Tris buffer, pH 8.0 (for menadione and 2-cyclohexen-1-one) or 50 mM ammonium acetate buffer, pH 7.5 (for 3-cyanocoumarin). For menadione, the standard and reaction products were derivatized with methoxyamine. Specifically, the standard and products were dried by rotary evaporation, resuspended in 20 μl pyridine containing 20 mg mL^-1^ methoxyamine hydrochloride, and incubated at 37°C for 1.5 h. To this solution, 20 μl of *N*-methyl-*N*-(trimethylsilyl)trifluoroacetamide (MSTFA) was added and the solution was incubated at 37°C for 1 h. For cyclohexenone, the standard and reaction products were derivatized by spiking the solution with 1 mM 2,4-dinitrophenylhydrazine and incubating the solution at 30°C for 2 h.

### LC/MS and GC/MS

The standard and reaction products of 3-cyanocoumarin were measured on an Agilent 6100 Series Single Quadrupole liquid chromatography-mass spectrometry (LC/MS) with diode array detector. Samples were separated on an Agilent Poroshell 120 EC-C18 column (2.7 μm, 2.1 × 100 mm). A gradient of two buffers, buffer A (0.1% formic acid in H_2_O) and buffer B (0.1% formic acid in acetonitrile), was applied as follows: 0–0.5 min, held at 10% B; 0.5–6.5 min, 10–60% B; 6.5–7 min, held at 90% B. A positive mode electron ionisation (EI) scan was undertaken, and in these conditions the molecular ion could not be detected as the loss of the cyano (CN) group was universal. The derivatized cyclohexenone standard and reactions products were determined on an Agilent 1290 Infinity/6550 quadrupole time-of-flight (Q-TOF) LC/MS system equipped with an Agilent Poroshell 120 EC-C18 2.1 × 50 mm 2.7 μm column. A gradient comprising two buffers, buffer A (20 mM ammonium acetate, pH 7.0) and buffer B (100% acetonitrile), was applied as follows: 0–1 min, held at 10% B; 1–10 min, 10–90% B. Positive mode electrospray ionisation (ESI) was utilized, and a scan from 50 to 300 *m/z* was conducted. The menadione standard, its reaction product, and their methoxime derivatives were detected on an Agilent 7010 gas chromatography-mass spectrometry (GC/MS) triple quadrupole system. Samples were separated on an Agilent 19091S 30 m × 250 μm × 0.25 μm HP-5 ms column over a gradient of 60–320°C at a rate of 7°C min^-1^, and a positive EI scan at 70 eV was conducted.

## Results

### F_420_H_2_-Dependent Reductases Reduce Structurally Diverse Cyclic and Polycyclic Compounds

**FIGURE 1:**
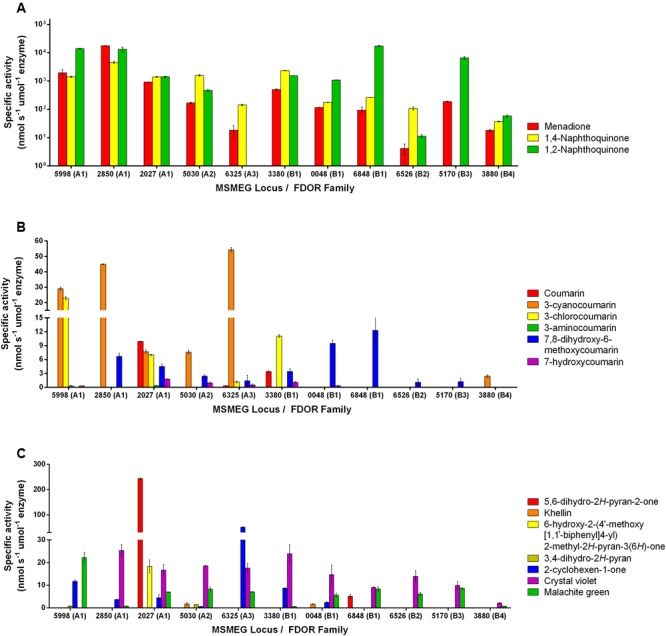
Substrate range of the F_420_H_2_-dependent reductases. The results show the specific activities of five FDOR-A and six FDOR-B enzymes with 16 organic compounds. Specific activities are shown with **(A)** quinones, **(B)** coumarins, and **(C)** pyrones, pyrans, cyclohexenones, and triarylmethanes. Error bars show standard deviations from three independent replicates. The reactivity of six of the enzymes with quinone and triarylmethane compounds was previously reported (Jirapanjawat et al., 2016). The structures of the substrates are shown in Supplementary Table S2. The 32 compounds tested that were not compatible with the FDORs are listed in Supplementary Table S3.

Eleven F_420_H_2_-dependent FDORs from *M. smegmatis* were expressed recombinantly and purified (Supplementary Table S1). We purified enzymes spanning multiple phylogenetically distinct subgroups, namely three enzymes each from the well-described FDOR-A1 and FDOR-B1 subgroups (Taylor et al., 2010; Lapalikar et al., 2012b; Ahmed et al., 2015), as well as representatives from five other subgroups (A2, A3, B1, B2, B3, B4; Ahmed et al., 2015). On the basis of previously reported data (Lapalikar et al., 2012b; Gurumurthy et al., 2013; Jirapanjawat et al., 2016), we determined the specific activities of the purified enzymes with 47 organic compounds following addition of the pre-reduced cofactor F_420_H_2_ (Supplementary Tables S2, S3). Of these, 16 compounds were enzymatically transformed. These compounds included fundamental monocyclic compounds, such as 3,4-dihydro-*2H*-pyran, 2-cyclohexen-1-one, and 5,6-dihydro-2*H*-pyran-2-one, as well as aromatic bicyclic and tricyclic compounds from the quinone, coumarin, and arylmethane chemical classes (Supplementary Table S2). Specific activities for the 16 substrates ranged from very low if reproducible for some compounds (e.g., <1 nmol s^-1^ μmol enzyme^-1^ for the pyran compound) to high for the quinones (e.g., >10^4^ nmol s^-1^ μmol enzyme^-1^ for 1,2-naphthoquinone) (**Figure 1**). Enzymes purified from the A1, A3, and B1 classes had the broadest and highest activities, with MSMEG_2027 (A1) proving catalytically compatible with all but two of the 16 substrates, whereas enzymes from the A2, B2, B3, and B4 classes had low activities with all non-quinone substrates.

Comparisons across the compounds tested for activity suggest that the presence of an electrophilic alkene is necessary for reduction to occur and implicates this group as the hydride acceptor. For example, activity was observed with pyrones that were α-substituted (e.g., 5,6-dihydro-*2H*-pyran-2-one; **Figure 1C**) but not γ-substituted (e.g., chelidonic acid; Supplementary Table S3). This is also supported by the finding that, whereas malachite green and crystal violet can be reduced, azure B cannot; while all three compounds contain triphenyl and *N,N*-dimethyliminium moieties, azure B lacks the central delocalized alkene group (Supplementary Tables S2, S3). Consistent with the proposal that the activated alkene is the hydride acceptor of F_420_H_2_-dependent reductases, enzymatic activity with coumarin derivatives was modulated by the nature of aromatic directing groups at the C3 position (**Figure 1**); moderate activities were observed with electron-withdrawing cyano and chloro groups, very low activities with an electron-donating amino group, and no activity with 3-hydroxycoumarin (**Figure 1B**). This suggests that electron-withdrawing groups render these compounds susceptible to nucleophilic attack by removing electron density from the π system. It is possible that differential interactions of these substrates with the substrate-binding pockets also contribute to the differences in the rates of reduction both between substrates and between enzymes.

For three of the compounds, we also measured specific activities with another independent assay that measured substrate reduction by HPLC in a cofactor-recycling system containing the Fgd (Supplementary Figure S1). While the relative activities between enzymes were comparable, initial reduction rates were generally higher in the cofactor-recycling systems and resulted in substrate conversions exceeding 90%.

### F_420_H_2_-Dependent Reductases Selectively Reduce Electrophilic Alkene Groups

We subsequently sought to understand the structural basis of how FDORs could reduce such diverse substrates. To do this, we used automated substrate docking to compare the binding of representative substrates to the high-resolution crystal structures of the highly promiscuous MSMEG_2720 (Ahmed et al., 2015) (A1) and the more specific MSMEG_6526 (Ahmed et al., 2015) (B2) enzyme. Compounds representing four major substrate classes were tested, namely menadione (quinone class), 3-cyanocoumarin (coumarin class), 2-cyclohexen-1-one (monocyclic compounds), and malachite green (arylmethane class). Consistent with the results of the activity assays (**Figure 1**), all substrates were predicted to be structurally compatible with MSMEG_2027, whereas only menadione and malachite green were predicted to specifically bind MSMEG_6526 (Supplementary Figure S2 and Table S4).

**FIGURE 2:**
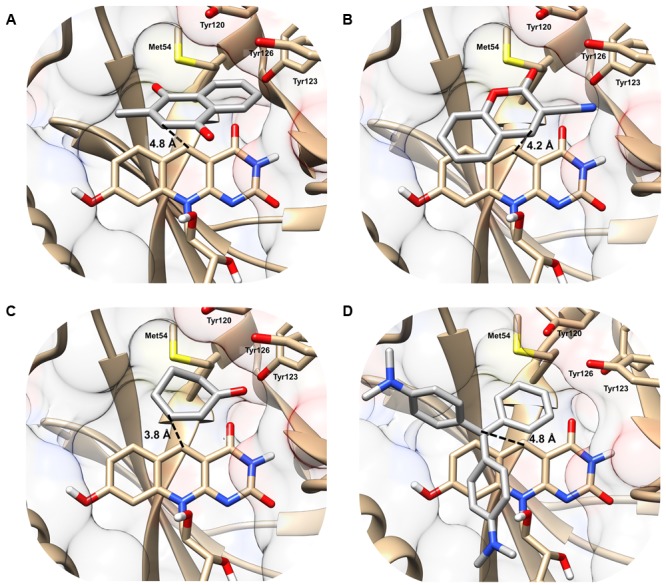
Structural basis of substrate activation by F_420_H_2_-dependent reductases. The secondary structure and surface rendering of the cofactor- and substrate-binding site of MSMEG_2027 are shown based on the 1.5 Å resolution crystal structure (PDB: 4Y9I) (Ahmed et al., 2015) of the enzyme. The structures are computationally docked with **(A)** menadione, **(B)** 3-cyanocoumarin, **(C)** 2-cyclohexen-1-one, and **(D)** malachite green. The distance between the proposed hydride donor (C5 of F_420_H^-^) and hydride acceptor (electrophilic carbon of the substrate) are shown. Residues within 5 Å of the substrate are shown. Docking results with the more specific F_420_H_2_-dependent reductase MSMEG_6526 are shown in Supplementary Figure S2 and are compared with MSMEG_2027 in Supplementary Table S4.

In the MSMEG_2027 models, substrates are accommodated in the large substrate-binding pocket adjacent to the cofactor-binding site (**Figure 2**). All four substrates are predicted to make extensive hydrophobic interactions with aromatic residues in the active site, including a triad of tyrosine residues (Tyr120, Tyr123, Tyr126) that have previously been shown to facilitate hydrophobic shielding during nitroimidazole activation (Mohamed et al., 2016b). There was also evidence of hydrophobic interactions between substrate and cofactor, including different degrees of π-stacking interactions with the isoalloxazine ring (**Figure 2**). Few polar interactions were predicted, except hydrogen bonds between the cyano group of 3-cyanocoumarin and the carbonyl oxygen of 2-cyclohexen-1-one with the hydroxyl group of Tyr123. The orientation of the substrates is likely to be realistic. For example, the binding poise of menadione suggests that menaquinone (the proposed physiological substrate of FDOR-A1 enzymes (Gurumurthy et al., 2013; Ahmed et al., 2015), which comprises a menadione headgroup and a polyisoprene tail) can be accommodated in the active site, given the polyisoprene tail at the C2 position is predicted to be oriented away from the active site.

The docking results indicate that hydride transfer can occur directly between cofactor and substrate within the hydrophobic environment of the MSMEG_2027 active site. Menadione, 3-cyanocoumarin, and 2-cyclohexen-1-one are predicted to be oriented such that their activated alkene groups are within 5 Å of the nucleophilic C5 center of F_420_H^-^ (**Figures 2A–C**). This suggests that, in line with the activity assays (**Figure 1**) and previously proposed mechanisms (Taylor et al., 2010; Lapalikar et al., 2012b; Ahmed et al., 2015; Mohamed et al., 2016b), catalysis will occur through nucleophilic attack of the C5 hydride to the electrophilic alkene. In the case of malachite green, the alkene moiety (C1 position) of the substrate is 4.2 Å away from C5 of the cofactor, whereas the *N,N*-dimethylamine and *N,N*-dimethyliminium moieties point toward the solvent phase (**Figure 2D**). Binding modes in which the *N,N*-dimethyliminium moiety was proximal to the cofactor caused steric occlusion. This again suggests that the alkene rather than imine moiety serves as the initial site of hydride transfer from F_420_H^-^. In comparison, docking with the less promiscuous MSMEG_6526 enzyme suggested that menadione and malachite green are only accommodated at orientations where the distance between the hydride donor and acceptor exceeds 7 Å, which will be suboptimal for catalysis (Supplementary Table S4). This reflects that MSMEG_6526 has a smaller binding pocket than MSMEG_2027 due to its larger flanking loops (Ahmed et al., 2015).

### F_420_H_2_-Dependent Reductases Mediate Substrate Reduction by Direct Hydride Transfer

**FIGURE 3:**
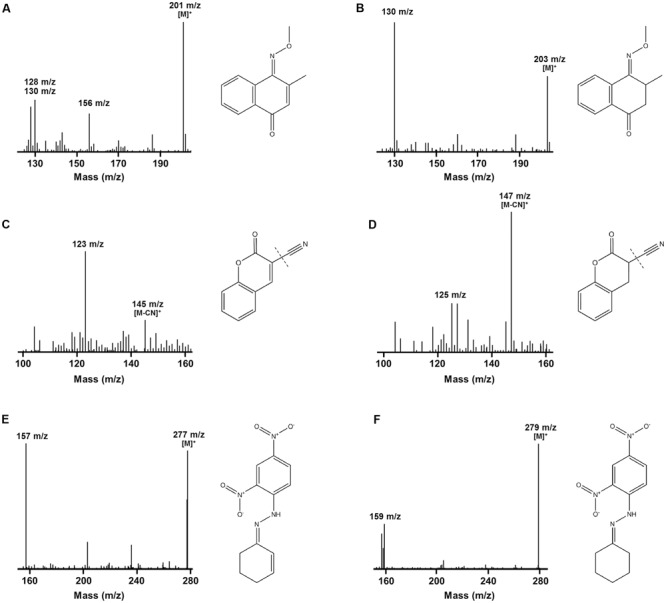
Mass spectra infer that F_420_H_2_-dependent reductases mediate hydrogenation of substrates. The spectra show the detection of substrate standards and reaction products following reduction with MSMEG_2027. GC/MS spectra of the single methoxime derivatives of the **(A)** menadione standard and **(B)** 2,3-dihydromenadione product. Mass spectra of the underivatized compounds are shown in Supplementary Figure S3. Keto–enol tautomerization is likely to result in menadiol formation under physiological conditions. LC/MS spectra of the **(C)** 3-cyanocoumarin standard and **(D)** 3-cyanochroman-2-one product. The cyano groups were ionized by in-source fragmentation. LC/MS spectra of the dinitrophenylhydrazone derivatives of the **(E)** 2-cyclohexen-1-one standard and **(F)** cyclohexanone product. A mass spectrum of the underivatized product could not be obtained. In all cases, corresponding compounds are shown to the right of the spectra. A mass spectrum showing the reduction of malachite green to leucomalachite green was previously published (Jirapanjawat et al., 2016).

We determined the mechanistic basis of substrate promiscuity among the F_420_H_2_-dependent reductases. To do so, we used mass spectrometry to determine the products formed by the reduction of three representative substrates. LC/MS and GC/MS studies demonstrated that, following incubation of menadione, 3-cyanocoumarin, and 2-cyclohexen-1-one with MSMEG_2027, each substrate peak increased by 2 *m/z* (**Figure 3**). This suggests that this enzyme catalyses the reduction of menadione (172 Da) to either menadiol or 2,3-dihydromenadione (both 174 Da) (Supplementary Figure S3), 3-cyanocoumarin (171 Da) to 3-cyanochroman-2-one (173 Da) (**Figures 3C,D**), and 2-cyclohexen-1-one (96 Da) to either 2-cyclohexen-1-ol or cyclohexanone (both 98 Da) (**Figures 3E,F**). This is consistent with previous observations that F_420_H_2_-dependent reductases mediate hydride transfer and subsequent protonation of their substrates (Taylor et al., 2010; Lapalikar et al., 2012b; Ahmed et al., 2015; Jirapanjawat et al., 2016; Mohamed et al., 2016b). In previous LC/MS studies, we demonstrated that malachite green (329 Da) was transformed by MSMEG_2027 to produce a decolorized product likely to be the protonated form of leucomalachite green (331 Da) (Jirapanjawat et al., 2016).

While these findings suggest F_420_H_2_-dependent reductases mediate hydride transfer from F_420_H_2_ to substrate, they do not resolve whether the site of attack is the alkene or carbonyl groups of the compounds. To resolve this, we derivatized the standard and reaction products of menadione with methoxyamine hydrochloride (carbonyl-specific) and MSTFA (alcohol-specific). GC/MS analysis of the reaction products revealed that reduction of menadione occurred exclusively *via* the alkene group (**Figures 3A,B**). Single and double methoxime derivatives of reduced menadione could be detected, indicating 2,3-dihydromenadione was formed as the major reaction product. No trimethylsilyl ester derivatives were formed under these conditions, underlining the absence of menadiol and other quinol products. Menadiol is nevertheless likely to form under physiological conditions through keto–enol tautomerism. In the case of cyclohexenone, the standard and reaction products were derivatized with 2,4-dinitrophenylhydrazine (carbonyl-specific), and analyzed by LC/MS. Analysis of product formation revealed the emergence of the hydrazone derivative of cyclohexanone, again indicating that reduction was mediated through the alkene (**Figures 3E,F**). Previous studies have inferred that coumarin reduction also occurs through the activated alkene group (Taylor et al., 2010; Lapalikar et al., 2012a,b).

## Discussion

**FIGURE 4:**
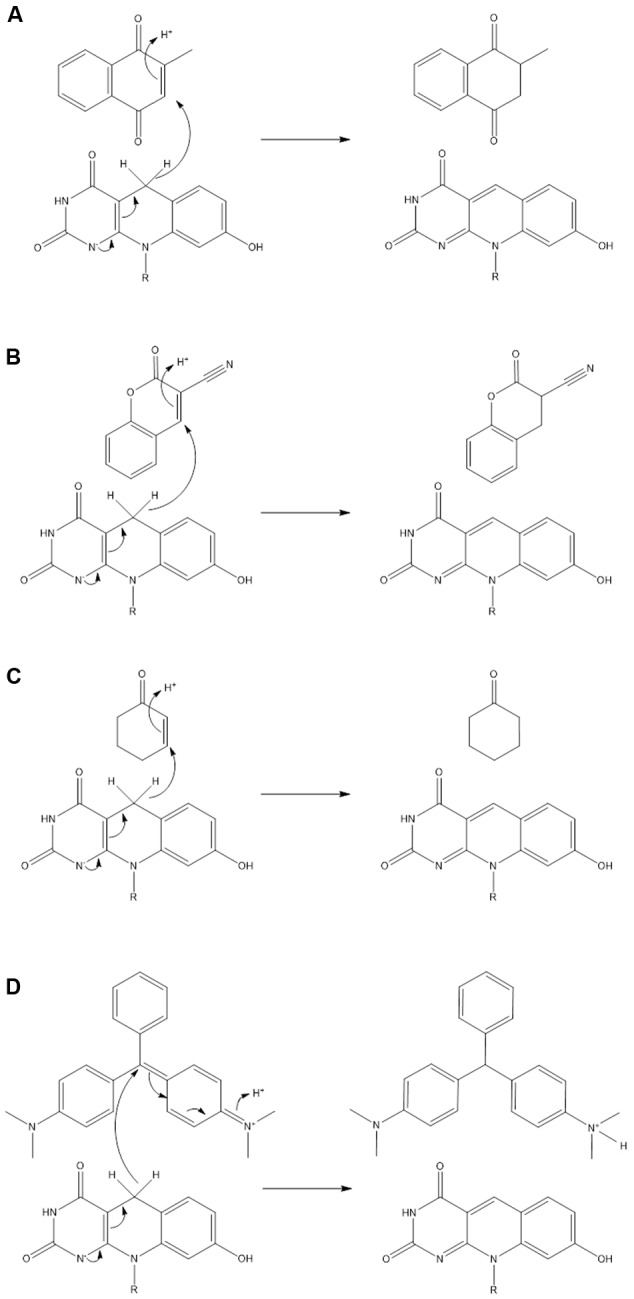
Unifying mechanism for the hydrogenation of representative substrates by F_420_H_2_-dependent reductases. Reaction mechanisms are proposed for **(A)** menadione, **(B)** 3-cyanocoumarin, **(C)** 2-cyclohexen-1-one, and **(D)** malachite green. On the basis of previous studies, it is predicted that the cofactor binds in its deprotonated state (F_420_H^-^) (Mohamed et al., 2016a) and that a hydroxonium ion serves as the proton donor for the substrate (Mohamed et al., 2016b).

In this study, we explored the potential of actinobacterial F_420_H_2_-dependent reductases as industrial biocatalysts. We show that mycobacterial FDORs use the electron donor F_420_H_2_ to hydrogenate diverse organic compounds at a wide range of rates. On the basis of these findings, we propose in **Figure 4** that all FDOR substrates studied can be reduced through a common hydrogenation mechanism: The cofactor binds the FDOR in its deprotonated state (F_420_H^-^; Mohamed et al., 2016a) and the substrate thereafter binds the adjacent pocket through hydrophobic interactions with aromatic residues and the cofactor. Alignment of the nucleophilic C5 center of F_420_H^-^ with the electrophilic alkene group of the substrate will promote direct hydride transfer. Subsequent steps will result in delocalization of electron charge and protonation of the substrate by a solvent-accessible hydroxonium ion (Mohamed et al., 2016b). The FDORs promote this mechanism in multiple ways: binding the substrate and cofactor in proximal sites; generating a hydrophobic environment that promotes hydride transfer; and facilitating protonation by binding hydroxonium ions through conserved tyrosine residues (Mohamed et al., 2016b). The overall mechanism of these enzymes is therefore equivalent to the old yellow enzymes (Stuermer et al., 2007), though the hydride and proton donors are distinct.

The hydrogenation mechanism proposed here is supported by our studies exploring the observed substrate range of the FDORs (**Figure 1**). We showed that, in line with findings about the substrate range of old yellow enzymes (Stuermer et al., 2007), the presence of an electrophilic alkene group was a prerequisite for reduction to occur and that rates were enhanced in electron-withdrawing conjugated systems. The outlined mechanism is also consistent with the results of the structural modeling (**Figure 2**) and mechanistic studies (**Figure 3** and Supplementary Figure S3) that identified the probable sites of hydride attack and inferred hydrogenated reaction products using four model substrates, menadione, 3-cyanocoumarin, 2-cyclohexen-1-one, and malachite green. Similar mechanisms have been proposed for other important reactions known to be mediated by F_420_H_2_-dependent reductases of the FDOR superfamily, namely activation of nitroimidazole prodrugs (Mohamed et al., 2016a,b), reduction of biliverdin to bilirubin (Ahmed et al., 2015, 2016), and the terminal step in the biosynthesis of tetracyclines (Wang et al., 2013). Our mass spectral analysis suggests that these mechanisms are regioselective, with hydride transfer only favorable to electrophilic alkene groups proximal to the nucleophilic C5 center. It will be necessary to extend studies to substrates that will produce prochiral products to determine whether this process also occurs stereoselectively, i.e., through *cis* or *trans* hydrogenation. The observation that substrate reduction is faster in the cofactor-recycling assays is also of interest, and suggests that there is a mechanism that enhances cofactor exchange between FDORs and Fgd (e.g., complex formation).

Our findings warrant the further exploration of F_420_H_2_-dependent FDORs in *in vitro* and *in vivo* biocatalytic processes. Their inherent substrate range, combined with their ease of heterologous overexpression and the presence of a viable cofactor-recycling system, suggests that these enzymes have promise in *in vitro* systems. There may be particular value in exploring the use of these enzymes for hydrogenating substrates incompatible with inorganic catalysts or old yellow enzymes (Stuermer et al., 2007; Clouthier and Pelletier, 2012). Particularly promising are the findings that enzymes in the FDOR superfamily mediate critical steps in the biosynthesis of tetracycline antibiotics (Wang et al., 2013) and the preliminary results that the membrane-bound FDOR-AA family can saturate linear fatty acid chains (Ahmed et al., 2015). However, at least two major innovations are needed if F_420_H_2_-dependent reductases are to be more widely developed: Firstly, given the observation that most substrates were reduced at low rates, the directed evolution of promising FDORs (e.g., MSMEG_2027) will be required to enhance their activities with desirable substrates. Secondly, new processes must be developed if F_420_ is to be cheaply and conveniently produced (Greening et al., 2016). It may be possible to engineer the production of this cofactor in recombinant systems, but this depends on the resolution of the complete F_420_ biosynthesis pathway. Alternatively, it is plausible to synthesize deazaflavin analogs that are catalytically compatible with F_420_H_2_-dependent reductases, which have previously been shown to exhibit cofactor promiscuity (Lapalikar et al., 2012a). There is more immediate promise in using these enzymes within actinobacterial hosts and recombinant systems to produce natural products or bioremediate contaminants. With the vast majority of F_420_-dependent oxidoreductases remaining functionally unannotated, it is expected that further study of these enzymes will reveal novel reactions of potential industrial and pharmaceutical relevance.

## Author Contributions

CG, AW, JO, MT, BN, CJ, TJ, CS, GP, RR, and BL designed experiments. TJ, CG, SA, BN, AW, MT, and BL performed experiments. CG, JO, CJ, AW, MT, RR, CS, GP, and BL supervised students. CG, AW, JO, BN, TJ, MT, CJ, and SA analyzed data. CG, TJ, AW, BN, and JO wrote the paper. Specific authors were responsible for the specific activity assays (TJ, CG, SA, BL, AW, MT, JO, CJ), substrate-docking experiments (CG, TJ, CJ), and analytical chemistry assays (BN, CG, TJ, AW).

## Conflict of Interest Statement

The authors declare that the research was conducted in the absence of any commercial or financial relationships that could be construed as a potential conflict of interest.
