# Supplementary material for: Mycobacterial F420H2-Dependent Reductases Promiscuously Reduce Diverse Compounds through a Common Mechanism
Source: Front Microbiol. 2017 May 31;8:1000. doi: 10.3389/fmicb.2017.01000 (PMC5449967; doi:10.3389/fmicb.2017.01000)

**Supporting Information**

**Table S1.** Enzyme subgroups, molar absorption coefficients, and amino acid sequences of the eleven F_420_H_2_-dependent reductases tested from *Mycobacterium smegmatis*.

| **Enzyme locus** | **Enzyme subgroup** | **Coefficient (M^-1^ cm^-1^)** | **Protein sequence** |  |
| --- | --- | --- | --- | --- |
| MSMEG_5998 | FDOR-A1 | 23950 | MADTSRPLNAKQLERLNAKSTGTLIKWMSRFQTFLFKTTNGKLGNKFLRGTEVGILTTIGRKSGEPRDTPLLFLQEGRRIVLVASQGGRATNPMWYLNLKANPKVTFQTRSEKLALVAREATDAERDEYWPKLDAMYPDFANYRSYTDRKIPIVICDPA | |
| MSMEG_2850 | FDOR-A1 | 16960 | MTDAELSPTDWVREQTERILEQGTTDGVHVLDRPIVLFTTTGAKSGKKRYVPLMRVEENGKYAMVASKGGDPKHPSWYFNVKANPTVSVQDGDKVLPDRTARELEGEEREHWWKLAVEAYPPYAEYQTKTDRLIPVFIVE | |
| MSMEG_2027 | FDOR-A1 | 30940 | MTDAELSPTDWVREQTERILEQGTTDGVHVLDRPIVLFTTTGAKSGKKRYVPLMRVEENGKYAMVASKGGDPKHPSWYFNVKANPTVSVQDGDKVLPDRTARELEGEEREHWWKLAVEAYPPYAEYQTKTDRLIPVFIVE | |
| MSMEG_5030 | FDOR-A2 | 32430 | MPWWERYIGLPLLLLHDKVYKATDGRIGHRIPGGPATLILHTVGAKTGQHRASSLAYARDGDDYLVVASKGGEPKAPGWYHNLKADPNVEINVGPKRLRATARAVFPDDPDFPRLWEIVNNMPGNKDRYIGYQKRTTRQIPVIVLTPVS | |
| MSMEG_6325 | FDOR-A3 | 44920 | MDDKLHGIPRVDLETRPRWKRDLAWWFGGKVLATARASAIWRKIAMPYEVPLIKATGGRARLSVGIPIAVLTSTGARSGKTRQTALAYFTDGDDVVLIASNYGQARHPGWYHNLRAHPECELYVGRRGGRFVAREVDGPQRDRLYALAASRLYPGWVAYEKRAEGVRRIPVLRLTPADP | |
| MSMEG_3380 | FDOR-B1 | 21430 | MVAVPEGYESLLERPLYGHLATVRPDGTPQVNAMWFAWDGEVLRFTHTTKRQKYRNIKANPAVAMSVIDPDNPYRYLEVRGLVEDIVPDPTGAFYLKLNDRYDGPLTEPPADKADRVIIVVRPTAFSKQ | |
| MSMEG_0048 | FDOR-B1 | 29450 | MGKNERTKIVMSDEEIAEFVERSRTATMATVLPDGRPHLVAMWYAVVDGEIWFETKAKSQKAVNLRRDPTVTVLIEDGHTYDTLRGVSIDGTAEIVDDPETLLRVGISVWERYTGPYTDEMRPFVDQMMNNRIAVRVVPGRTRSWDHRKLGMPAMPLGGSTAQYLNS | |
| MSMEG_6848 | FDOR-B1 | 27515 | MGVSPARLRQVLDAPVFGTVATIDPDGAPQQSVVWVGRDGDDVLFAVATGSRKERNLRRDPRVSILLSPPDEPYTYAVIHGKATLHTEGGHQLRDALAVKYTGKTYAEGNADAAARYGDVAMTVVRVTPERTVGRL | |
| MSMEG_6526 | FDOR-B2 | 34950 | MAEFDAVTAFADAPAAVLSTLNADGAPHLVPVVFAVHVPHVEGQPARIYTAVDAKRKTTRNLRRLANIDRDSRVSLLVDHYSDDWTQLWWVRADGVATTHHSGDEVATGYALLRAKYHQYERVSLDGPVISVEVSRWASWQA | |
| MSMEG_5170 | FDOR-B3 | 16960 | MGRQVFDDKLLALICNNSLGVLATIKQDGRPQLSNVSYHFDPRAQTFQVSITEPRAKTRNLRRDPRASIHVSSDDGWAYAVAEGDAILTPPAASTHDDTVEGLIALYRNISGEHPDWDEFRQAMVDDRRVLMTLPITHVYGMPPGMR | |
| MSMEG_3880 | FDOR-B4 | 8480 | MAASRGKATTRLTTDALAFLTERHLAMLTTLRSDGSPHVVAVGFTFDPKTHIARVITTGGSQKAVNAQERGVAVLSQVDGARWLSLEGKSTVSSDPDAVRDAELRYAQRYRTPRVNPRRVVIEVRIERVLGSSELLDRS | |
|  |  |  |  | |

**Table S2.** Chemical structures of the 16 compounds tested that were reduced by F_420_H_2_-dependent FDORs.

| **Compound** | **Compound class** | **Structure** |
| --- | --- | --- |
| Menadione | Quinones |  |
| 1,4-napthoquinone | Quinones |  |
| 1,2-napthoquinone | Quinones |  |
| Coumarin | Coumarins |  |
| 3-cyanocoumarin | Coumarins |  |
| 3-chlorocoumarin | Coumarins |  |
| 3-aminocoumarin | Coumarins |  |
| 7,8-dihydroxy-6-methoxycoumarin | Coumarins |  |
|  |  |  |
| 7-hydroxycoumarin | Coumarins |  |
| 5,6-dihydro-2H-pyran-2-one | Pyrones |  |
| Khellin | Pyrones |  |
| 6-hydroxy-2-(4’-methoxy[1,1’-biphenyl]4-yl)-2-methyl-2*H*-pyran-3(6*H*)-one | Pyrones |  |
| 3,4-dihydro-2*H*-pyran | Pyrans |  |
| 2-cyclohexen-1-one | Cyclohexenones |  |
| Crystal violet | Arylmethanes |  |
| Malachite green | Arylmethanes |  |

**Table S3.** Chemical structures of the 31 compounds tested that were catalytically incompatible with F_420_H_2_-dependent FDORs.

| **Compound** | **Compound class** | **Structure** |
| --- | --- | --- |
| Anthraquinone | Quinones |  |
| 1,4-benzoquinone | Quinones |  |
| 3-hydroxycoumarin | Coumarins |  |
| 4-hydroxycoumarin | Coumarins |  |
| 4-methoxy-6-methyl-2H-pyran-2-one | Pyrones |  |
| 3-hydroxy-2,6-bis(hydroxymethyl)-4H-pyran-4-one | Pyrones |  |
| EHT 1864 | Pyrones |  |
|  |  |  |
| Chelidonic acid | Pyrones |  |
| Phenol red | Triarylmethanes |  |
| Benzonitrile | Benzene derivatives |  |
| 2-aminobenzonitrile | Benzene derivatives |  |
| 2,4-difluorobenzonitrile | Benzene derivatives |  |
| Cinnamic acid | Benzene derivatives |  |
| 3-phenoxybenzaldehyde | Benzene derivatives |  |
| Terephthalic acid | Benzene derivatives |  |
| Indole-3-carboxylate | Indoles |  |
|  |  |  |
| Potassium indole-6-trifluoroborate | Indoles |  |
| Cytosine | Pyridines |  |
| Thymine | Pyridines |  |
| Guanine | Purines |  |
| Adenine | Purines |  |
| Xanthine | Purines |  |
|  |  |  |
| Hypoxanthine | Purines |  |
|  |  |  |
| Caffeine | Purines |  |
| Adenosine | Purines |  |
|  |  |  |
| 5-nitroimidazole | Imidazoles |  |
|  |  |  |
| Benzimidazole | Imidazoles |  |
|  |  |  |
| 2-aminobenzimidazole | Imidazoles |  |
|  |  |  |
| Paraquat dichloride | Other |  |
|  |  |  |
| Azure B | Other |  |
|  |  |  |
| Thiamine | Other |  |

**Table S4.** Comparison of the donor-acceptor distances inferred from molecular docking studies and specific activities calculated from enzyme assays.

|  | **Donor-acceptor distance (Å)** | **Specific activity (nmol min^-1^ (μmol enzyme)^-1^)** |
| --- | --- | --- |
| **MSMEG_2027 (A1)** |  |  |
| Menadione | 4.8 | 917 ± 10 |
| 3-cyanocoumarin | 4.2 | 7.7 ± 0.4 |
| 2-cyclohexen-1-one | 3.8 | 4.6 ± 1.5 |
| Malachite green | 4.8 | 7.0 ± 0.2 |
|  |  |  |
| **MSMEG_6526 (B2)** |  |  |
| Menadione | 7.5 | 4.1 ± 1.8 |
| 3-cyanocoumarin | N/A | 0 |
| 2-cyclohexen-1-one | N/A | 0 |
| Malachite green | 6.9 | 6.1 ± 0.8 |

**Figure S1.** Comparison of the in rates of F_420_H_2_-dependent reductase activity observed in two different assay setups. The specific activities are shown for (a) 1,4-napthoquinone, (b) 3-cyanocoumarin, and (c) 5,6-dihydro-2*H*-pyran-2-one based on initial reduction rates. Yellow bars show reduction rates with pre-reduced F_420_H_2_, whereas red bars show rates in a cofactor-recycling system containing F_420_ and Fgd. Error bars show standard deviations from three independent replicates.





**Figure S2.** Structural basis of substrate activation by MSMEG_6526. The secondary structure and surface rendering of the cofactor- and substrate-binding site of MSMEG_6526 are shown based on the 1.7 Å resolution crystal structure (PDB: 4ZKY) of the enzyme (Ahmed et al., 2015). The structures are computationally docked with (a) menadione and (b) malachite green. The distance between the proposed hydride donor (C5 of F_420_H^-^) and hydride acceptor (electrophilic carbon of the substrate) are shown. Residues within 5 ‎Å of the substrate are shown. Computational docking suggested that 3-cyanocoumarin and 2-cyclohexen-1-one cannot specifically bind to this enzyme.


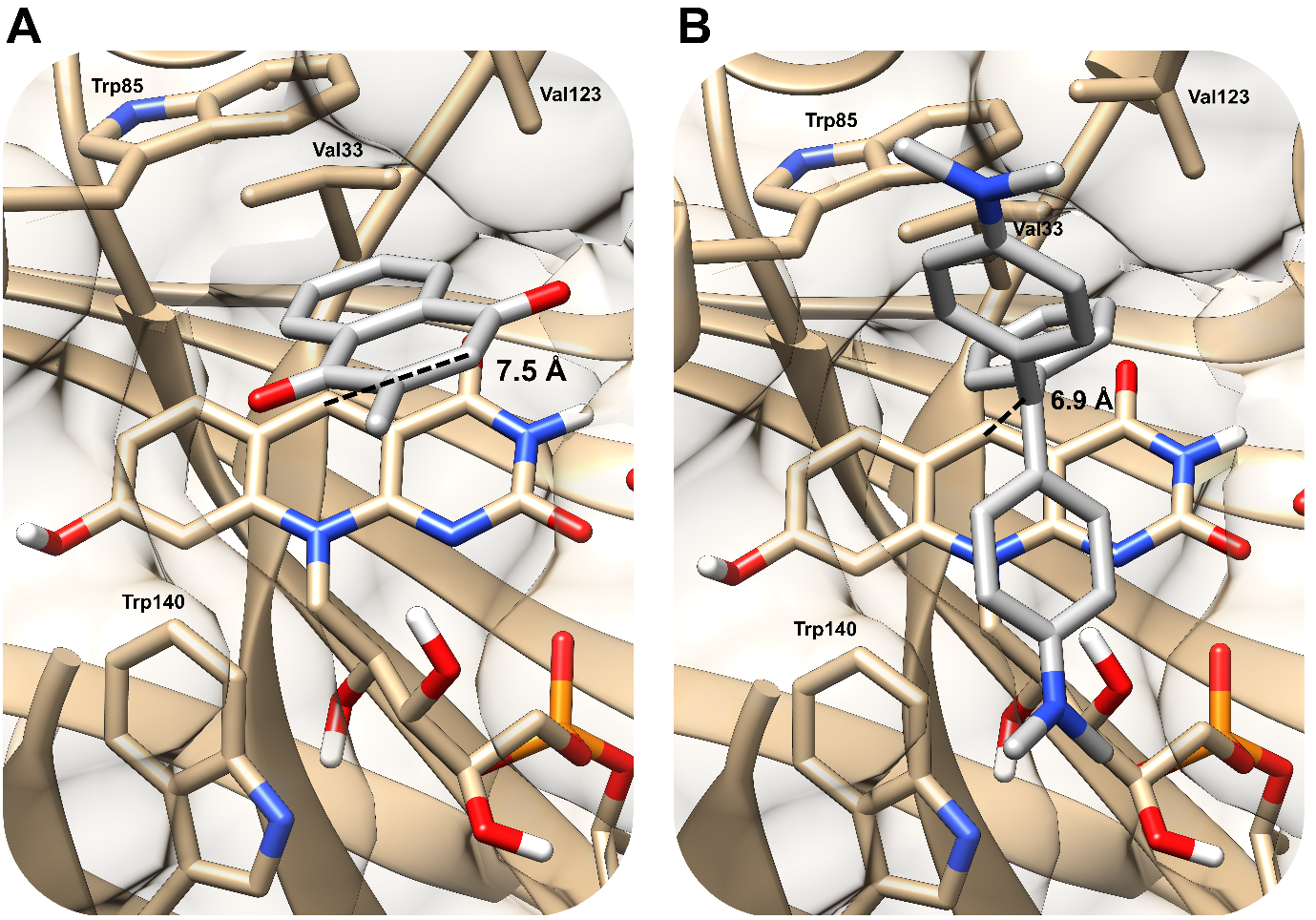


**Figure S3.** GC/MS detection of menadione, reaction products, and their methoxime derivatives. The mass spectra shown are of (a) menadione standard, (b) 2,3-dihydromenadione reaction product, (c) single methoxime derivative of menadione, (d) single methoxime derivative of 2,3-dihydromenadione, and (e) double methoxime derivative of 2,3-dihydromenadione. The corresponding compounds are shown to the right of the spectra. It is likely that 2,3-dihydromenadione will be converted to menadiol under physiological conditions through keto-enol tautomerism.


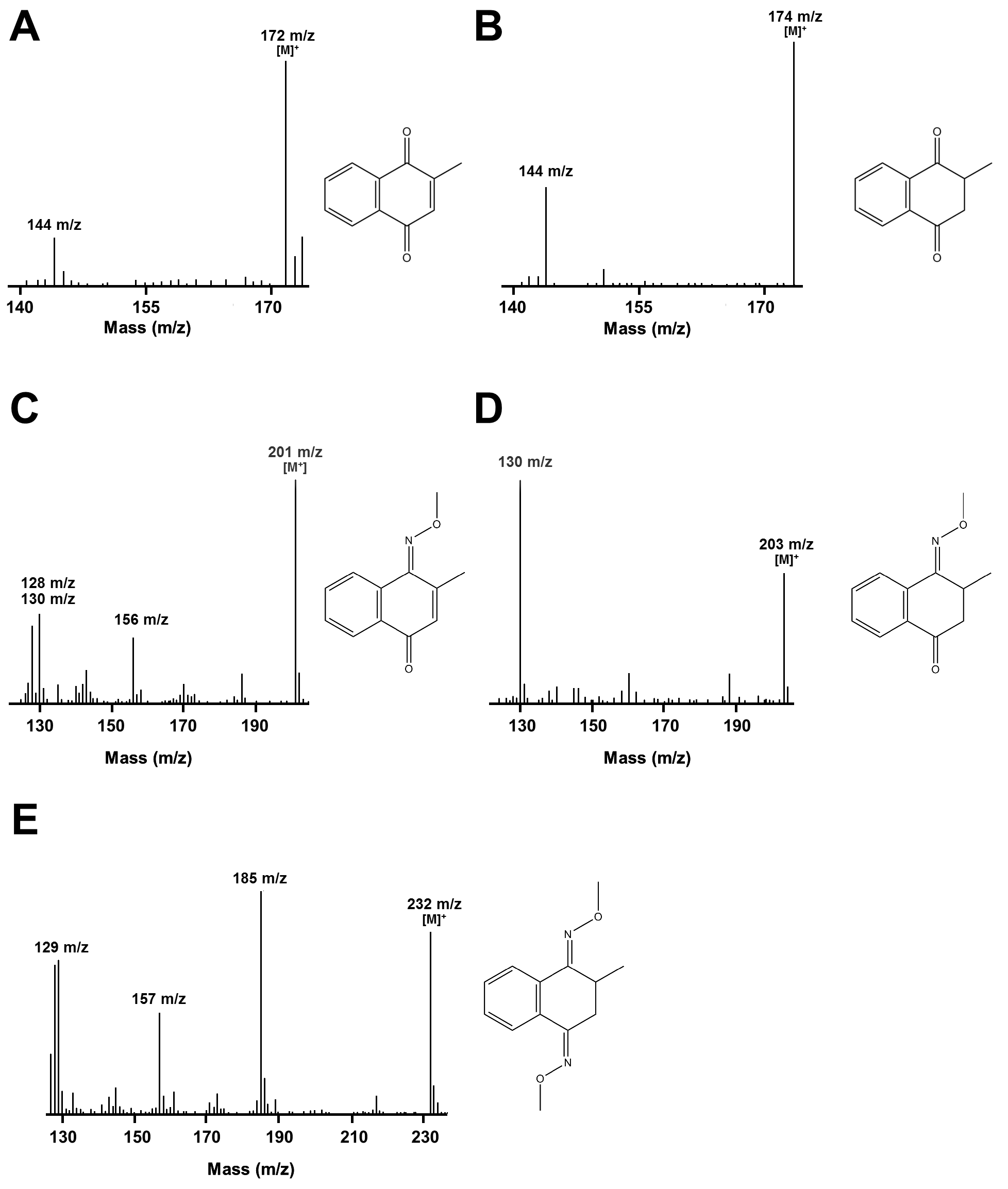

Supplement: Supplementary file 1 [file Data_Sheet_1.DOCX]
